# Supplementary material for: Integrating Novel and Classical Prognostic Factors in Locally Advanced Cervical Cancer: A Machine Learning-Based Predictive Model (ESTHER Study)
Source: J Pers Med. 2025 Apr 15;15(4):153. doi: 10.3390/jpm15040153 (PMC12029003; doi:10.3390/jpm15040153)
Supplement: Supplementary file 1 [file jpm-15-00153-s001.zip › jpm-3535890-supplementary.pdf]

SUPPLEMENTARY MATERIALS S1

Patients related data

- age
- body mass index (BMI, calculated as weight (Kg) divided by the square of height (m))
- hemoglobin level (Hg, in g/100 mL)

Tumor related data

- histological type (squamous cell carcinoma, adenocarcinoma)
- Federation of Gynecology and Obstetrics (FIGO) stage, based on the 2018 version
- clinical tumor stage
- clinical nodal stage
- maximum tumor diameter.

Treatment Related Data

- radiotherapy technique (3-D conformal radiotherapy, intensity modulated radiotherapy, or volumetric modulated arc therapy)
- EBRT dose (Gy) and fractionation on the pelvis
- brachytherapy boost dose (Gy)
- total tumor dose (Gy)
- overall treatment time (EBRT plus BRT, days).

Inflammatory Indices

- neutrophil-lymphocyte ratio (NLR)
- platelet-lymphocyte ratio (PLR)
- monocyte-to-lymphocyte ratio (MLR)
- systemic immune inflammation index (SII, calculated as platelet × neutrophil/lymphocyte)
- leukocyte-to-lymphocyte ratio (LLR)
- combination of platelet (PLT) count and NLR (COP-NLR, scored as follows: 0: NLR < 3 and PLT < 300 × 10^9/L; 1: NLR > 3 or PLT > 300 × 10^9/L; 2: NLR > 3 and PLT > 300 × 10^9/L)
- aspartate aminotransferase/platelet count ratio index (APRI, calculated as [aspartate aminotransferase {IU/L}/upper limit normal/PLT {×10^9/L}] × 100)
- aspartate aminotransferase-to-lymphocyte ratio index (ALRI, calculated as aspartate aminotransferase value [U/L]/lymphocyte count [× 10^9/L])
- systemic inflammatory response index (SIRI, calculated as neutrophil × monocyte/lymphocyte)
- aspartate transaminase to neutrophil ratio index (ANRI, calculated as aspartate aminotransferase/neutrophils).

All IIs referred to routine blood exams performed before CRT.

Body composition parameters

- BMI calculated as the ratio of an individual weight in kilograms to the square of their height in meters (BMI = weight in kg/(height in m)^2) and stratified into four categories (underweight—BMI <18.5 kg/m^2; normal weight—18.5 to 24.9 kg/m^2; overweight—25 < BMI < 29.9 kg/m^2; and obesity—BMI ≥ 30 kg/m^2)

- Sarcopenia calculated as follows: the skeletal muscle area (SMA) was defined on pre-treatment CT scans at the level of the third lumbar vertebra (L3); all relevant muscle structures were contoured at that level; the SMA was divided by body surface area, obtaining the skeletal muscle index (SMI); sarcopenic patients were defined by using a cut-off established by calculating the median skeletal muscle index (SMI) value minus 2 standard deviations; the cut-off from Prado and Martin were derived to establish if the sarcopenic condition was present or not [**34,35**].

- Sarcopenic obesity defined by assessing the prevalence of SP alongside the co-presence of a BMI ≥ 30 kg/m^2, based on the criteria set forth by Prado et al. [**34**]
